# X-ray-free ultrasound-guided versus fluoroscopy-guided percutaneous nephrolithotomy: a comparative study with historical control

**DOI:** 10.1007/s11255-020-02577-w

**Published:** 2020-07-24

**Authors:** Ponco Birowo, Putu Angga Risky Raharja, Harun Wijanarko Kusuma Putra, Reginald Rustandi, Widi Atmoko, Nur Rasyid

**Affiliations:** Department of Urology, Faculty of Medicine, Cipto Mangunkusumo Hospital, University of Indonesia, Jakarta, 10430 Indonesia

**Keywords:** Percutaneous nephrolithotomy, Supine, Ultrasound-guided, X-ray-free

## Abstract

**Purpose:**

To compare the outcomes and complications of supine X-ray-free ultrasound-guided percutaneous nephrolithotomy (XG-PCNL) with fluoroscopy-guided (FG)-PCNL in both prone and supine positions.

**Methods:**

This was a comparative study that included a prospective cohort and historical control groups. This study analysed 40 consecutive patients who undergone supine XG-PCNL between October 2019 and March 2020. The control groups were composed of historical control formed from the last 40 consecutive patients who underwent FG-PCNL in both supine and prone positions from our PCNL database from January 2018 and September 2019. Patients’ demographics, stone characteristics and intraoperative and postoperative outcomes were compared.

**Results:**

A total of 120 patients were classified into the supine XG-PCNL, supine FG-PCNL, and prone FG-PCNL groups (each *N* = 40). They had similar baseline characteristics and initial stone burden. The supine XG-PCNL group had higher puncture attempts, nephrostomy tube placement, and longer surgery duration than both the supine and prone FG-PCNL groups. However, the stone-free rate was similar in all groups (85%, supine XG-PCNL; 72.5%, supine FG-PCNL; 77.5% prone FG-PCNL; *p* = 0.39). No significant difference was found in the complication rate and length of stay among the three groups.

**Conclusion:**

Supine XG-PCNL is an alternative to both supine and prone FG-PCNL with similar efficacy and complication rates for kidney stone patients. This could be a good alternative to urological centres with no access to fluoroscopy.

## Introduction

Percutaneous nephrolithotomy (PCNL) is currently the leading minimally invasive procedure to remove large (i.e., > 20 mm) or complex kidney stones [1,2]. PCNL is conventionally assisted by fluoroscopy, however, it may present radiation exposure risk to both the patient and surgical team [3]. Thus, X-ray-free ultrasound-guided (XG)-PCNL was developed with the focus on decreasing radiation risks while sustaining real-time control during percutaneous access [4]. The use of XG-PCNL could also increase the number of provided PCNL procedures especially in the peripheral area, given the higher rate of ultrasound device availability in most peripheral hospitals. The total cost for every case of XG-PCNL was also approximately 30% less than the fluoroscopy-guided (FG)-PCNL [5].

Furthermore, the prone position has been the preferred method for establishing percutaneous access [6,7]. However, the prone position is related with various drawbacks, such as surgical, anaesthetic, and logistical [7]. Therefore, the supine PCNL has been introduced to simplify the procedure and overcome those disadvantages. Supine PCNL has shorter operation duration and less frequent ventilatory and circulatory complications than prone PCNL [8]. Supine XG-PNCL has been reported to be a safe, feasible, and affordable procedure [4]. However, direct comparisons between supine XG-PCNL with FG-PCNL were still lacking in the current literatures. Although safe and effective, no study has reported about kidney dilatation using Alken metal telescoping dilators in XG-PCNL. Zhou et al. adapted balloon dilatation in their XG-PCNL procedures [9]. Thus, in this study, we decided to adapt the novel technique of supine XG-PCNL using Alken metal telescoping dilators for kidney dilatation and compared the outcomes and complications of supine XG-PCNL with FG-PCNL in both prone and supine positions.

## Methods

### Study population

This comparative study included a prospective cohort and historical control groups. Between October 2019 and March 2020, data of 40 consecutive patients who underwent supine XG-PCNL at our centre were prospectively collected. Informed consents were obtained from all patients. In this study, the prospective supine XG-PCNL group was compared with the PCNL database of patients who underwent supine and prone FG-PCNL at our centres between January 2018 and September 2019. The latest consecutive 40 patients who underwent both supine and prone FG-PCNL who fulfilled the criteria were selected as control groups. The study design was approved by our institutional review board (The Ethics Committee of the Faculty of Medicine, University of Indonesia; Ethical Approval number KET-442/UN2.F1/ETIK/PPM.00.02/2020).

### Inclusion and exclusion criteria

All patients with caliceal, pelvic, and upper ureteral stones with stone burden of ≥ 20 mm were included. Patients with uncorrected coagulopathy, congenital kidney anomalies, different PCNL techniques (mini PCNL, micro PCNL, or ultra mini PCNL), and intraoperative conversion to fluoroscopy were excluded from this study. Since supine XG-PCNL with Alken metal telescoping dilators was still a new experience for our centre, there were 6 patients who had intraoperative conversion to fluoroscopy. The conversions were caused by difficulty in obtaining percutaneous access during either puncture or tract dilatation. Their data were excluded from this study.

### Procedures

All participants underwent the same laboratory tests including blood routine, urine routine, and renal function tests prior to surgery. Preoperative computed tomography (CT) urography was routinely performed to evaluate the stone location, kidney anatomy, and position of surrounding important structures. Stone burden was calculated using the cumulative stone diameter. It was measured by combining the largest diameter of each stone in all planes [10].

All PCNL procedures were accomplished by a team of endourologists consisting of two different main surgeons (P.B. and W.A.). The XG-PCNL was performed in supine (Galdakao-modified Valdivia) position [11]. While in FG-PCNL groups, the positions were either prone or supine (Galdakao-modified Valdivia) as decided by the preference of main surgeons. Both surgeons were qualified to perform PCNL in both positions. All patients received preoperative prophylactic antibiotics. Preoperative antibiotics therapy based on urine culture results were given if there was any urinary tract infection. The PCNL procedure was performed under general or spinal anaesthesia. For ureteral access, retrograde open-end ureteral catheter (5 Fr/70 cm) was inserted. The ureteral catheter was used for injection of normal saline or contrast agent. Normal saline injection through continuous pump would dilate the collecting system, enabling artificial hydronephrosis and facilitating needle puncture, especially in the XG-PCNL group.

Percutaneous renal access was accomplished using a 20-cm puncture needle (1.3 mm/17.5 G). In the XG-PCNL group, the target calyx selection was identified prior to the operation based on the stone location and surgeon preference. Successful puncture was confirmed with urine flow from the puncture needle. Under ultrasound guidance, a guidewire (0.035-inch J-shaped stiff-guidewire) was advanced into the collecting system. The needle was then withdrawn. Fascial dilatation was performed with 8-Fr, 10-Fr, and 12-Fr fascial dilators. Alken metal telescoping dilators (6 Fr × 30 Fr) were used for kidney dilatation. Alken metal telescoping dilators were used because of their reusable properties and thus being more economical. Urine flow from the dilators confirmed that we had reached the collecting system. A 28–30-Fr/17-cm Amplatz sheath was then pushed on into the collecting system. In the XG-PCNL group, ultrasonography was used solely in all procedures, that is, evaluation of the kidney and stone, assistance of kidney puncture, and tract dilatation. In the FG-PCNL group, all those steps were performed under fluoroscopy guidance.

A 30-Fr rigid nephroscope was used during the PCNL procedure. Stone fragmentation was performed using a 3.4-Fr pneumatic lithotripter, 3.78-Fr shock-pulse lithotripter, or a combination of both. Stone forceps was used to evacuate the stone fragments. In the XG-PCNL group, both ultrasonography and nephroscopy were used to identify residual stones, infundibular laceration, or extravasation of urine. In the FG-PCNL group, those procedures were done under fluoroscopic guidance. Upon conclusion of the PCNL procedure, nephrostomy tube, double J (DJ) stent, or externalised ureteral catheter were placed based on any significant bleeding, residual stone fragments, or debris. Some patients had both nephrostomy tube and DJ stent.

### Evaluations

In this study, we compared the demographic parameters, stone characteristics, and operative and postoperative outcomes between supine XG-PCNL and FG-PCNL in the prone and supine positions. All patients had postoperative kidney-ureter-bladder (KUB) photo determine the stone-free status. Several patients with significant residual fragments or radiolucent stones also had postoperative CT scan. KUB photo could missed residual stone fragments ≤ 4 mm, [12] however patients with residual stone fragments ≤ 4 mm were clinically insignificant and considered to be stone-free in this study.

### Statistical analysis

Data were analysed using SPSS ver. 24.0 (SPSS Inc., Chicago, IL, USA). Data are shown as the mean (SD) and number (percentage) based on the type of data. Outcomes of the three groups were compared using the One-way analysis of variance test for continuous variables with normal distributions, and Kruskal–Wallis test was used if data were not normally distributed. Statistical comparisons of qualitative variables were done using either Chi square test or Fisher’s exact test. A *p* value < 0.05 was considered statistically significant.

## Results

A total of 120 patients were classified into the supine XG-PCNL, supine FG-PCNL, and prone FG-PCNL groups. Each group consisted of 40 patients, and their baseline demographic characteristics are presented in Table 1. The major parameters among the groups were similar at baseline. A higher number of patients in the supine XG-PCNL group (50%) had hydronephrosis, but this number was not statistically significant compared with both supine and prone FG-PCNL groups (32.5% and 46%, *p* = 0.64). The initial stone burden were also comparable among the groups (*p* = 0.16).

**Table 1:** Baseline patient demographics

Variable	Supine XG-PCNL (*N* = 40)	Supine FG-PCNL (*N* = 40)	Prone FG-PCNL (*N* = 40)	*p *value
Age (year)	49.2 ± 17.0	53.9 ± 11.0	54.7 ± 9.6	0.13
Sex				0.24
Male	17 (42.5%)	23 (57.5%)	24 (60.0%)	
Female	23 (57.5%)	17 (42.5%)	16 (40.0%)	
Body mass index (BMI) (kg/m^2^)	25.3 ± 5.4	26.0 ± 4.5	24.0 ± 4.8	0.19
Multiple stone				0.27
No	24 (60.0%)	17 (42.5%)	19 (47.5%)	
Yes	16 (40.0%)	23 (57.5%)	21 (52.5%)	
Classification of stone				0.15
Non-staghorn	14 (35.0%)	21 (52.5%)	22 (55.0%)	
Partial or complete staghorn	26 (65.0%)	19 (47.5%)	18 (45.0%)	
Hydronephrosis				0.64
None	20 (50.0%)	27 (67.5%)	22 (55.0%)	
Grade I	1 (2.5%)	1 (2.5%)	2 (5.0%)	
Grade II	2 (5.0%)	3 (7.5%)	5 (12.5%)	
Grade III	8 (20.0%)	4 (10.0%)	6 (15.0%)	
Grade IV	9 (22.5%)	5 (12.5%)	5 (12.5%)	
Previous stone surgery				0.44
No	27 (67.5%)	30 (75.0%)	32 (80.0%)	
Yes	13 (32.5%)	10 (25.0%)	8 (20.0%)	
Side of stone				0.35
Right	16 (40.0%)	21 (52.5%)	16 (40.0%)	
Left	24 (60.0%)	19 (47.5%)	24 (60.0%)	
Initial stone burden (mm)	27.5 ± 18.6	33.4 ± 20.2	35.1 ± 17.0	0.16
Preoperative haemoglobin (g/dL)	12.0 ± 2.2	12.7 ± 2.2	12.6 ± 1.7	0.30
Urinary tract infection				0.70
No	8 (20.0%)	10 (25.0%)	7 (17.5%)	
Yes	32 (80.0%)	30 (75.0%)	33 (82.5%)	

The operative outcomes are shown in Table 2. The supine XG-PCNL group had significantly higher puncture frequency (2.0 ± 1.7 times) than both supine FG-PCNL (1.3 ± 0.5 times; *p* < 0.01) and prone FG-PCNL (1.1 ± 0.2 times *p* < 0.01) groups. The supine XG PCNL group had significantly more upper and mid-pole punctures (25% and 42.5%, respectively) than both the supine FG-PCNL (7.5% and 5%, respectively) and prone FG-PCNL (10% and 10%, respectively) groups. The surgery duration was also significantly longer in the supine XG-PCNL group (121.5 ± 54.5 min) than in both supine and prone FG-PCNL groups (89.4 ± 30.0 min and 97.5 ± 30.7 min; *p* = 0.01). Intraoperative blood loss volumes were comparable among the three procedures. A statistically higher number of patients had nephrostomy tube and DJ stent after the procedure in the supine XG-PCNL group (42.5%) than in both supine FG-PCNL (12.5%) and prone FG-PCNL (10%) groups.

**Table 2:** Comparison of operative outcomes

Variable	Supine XG-PCNL (*N* = 40)	Supine FG-PCNL (*N* = 40)	Prone FG-PCNL (*N* = 40)	*p* value
Aneshesia				0.43
Spinal	37 (92.5%)	39 (97.5%)	39 (97.5%)	
General	3 (7.5%)	1 (2.5%)	1 (2.5%)	
Puncture frequency (times)	2.0 ± 1.7	1.3 ± 0.5	1.1 ± 0.2	** < 0.01**
Site of puncture				** < 0.01**
Upper pole	10 (25.0%)	3 (7.5%)	4 (10.0%)	
Mid pole	17 (42.5%)	2 (5.0%)	4 (10.0%)	
Lower pole	13 (32.5%)	35 (87.5%)	32 (80.0%)	
Surgery duration (min)	121.5 ± 54.5	89.4 ± 30.9	95.0 ± 30.8	**0.01**
Blood loss (mL)	177.1 ± 230.8	213.3 ± 210.7	174.4 ± 221.2	0.68
Post-procedural stenting				** < 0.01**
Externalized ureteral catheter	12 (30.0%)	15 (37.5%)	14 (35.0%)	
Double J stent	11 (27.5%)	20 (50.0%)	22 (55.0%)	
Double J stent + nephrostomy	17 (42.5%)	5 (12.5%)	4 (10.0%)	

The postoperative outcomes in our study are summarised in Table 3. We defined stone-free status as insignificant residual stone fragments with size ≤ 4 mm on postoperative imaging using plain abdominal X-ray or CT scan. The stone-free rate of the supine XG-PCNL was 85%. Although higher, this number was not statistically significant when compared in both supine and prone FG-PCNL groups (66% and 76%; *p* = 0.39). The stone burden decrements were similar among the groups, with decrement of more than 90% compared with the initial stone burden on all groups (*p* = 0.97). No significant complications were reported after the PCNL procedure on all groups. The modified Clavien-Dindo system was used to classify the complications of the three procedures (Table 4). The difference in postoperative blood transfusion rate and postoperative fever among the groups were statistically not significant. The length of stay after the PCNL procedure in the supine XG-PCNL group was 3.0 ± 1.4 days and comparable with those in both the supine FG-PCNL and prone FG-PCNL groups (3.2 ± 2.0 days and 2.7 ± 0.8 days; *p* = 0.37).

**Table 3:** Comparison of post-operative outcomes

Variable	Supine XG-PCNL (*N* = 40)	Supine FG-PCNL (*N* = 40)	Prone FG-PCNL (*N* = 40)	*p* value
Stone free status				0.39
Yes	34 (85.0%)	29 (72.5%)	31 (77.5%)	
No	6 (15.0%)	11 (27.5%)	9 (22.5%)	
Stone burden decrement (%)	91.8 ± 22.4	90.1 ± 16.9	92.1 ± 18.1	0.97
Postoperative haemoglobin (g/dL)	11.2 ± 1.5	11.9 ± 1.8	11.6 ± 1.7	0.19
Postoperative haemoglobin drop (%)	5.1 ± 10.9	4.7 ± 12.0	8.2 ± 7.4	0.26
Length of stay (days)	3.0 ± 1.4	3.2 ± 2.0	2.7 ± 0.8	0.37

**Table 4:** Complications classified by a modified Clavien–Dindo system

Variable	Supine XG-PCNL (*N* = 40)	Supine FG-PCNL (*N* = 40)	Prone FG-PCNL (*N* = 40)	*p* value
Grade I				0.66
Postoperative fever	8 (20.0%)	5 (12.5%)	7 (17.5%)	
Grade II				0.63
Blood transfusion	10 (25.0%)	7 (17.5%)	7 (17.5%)	

## Discussion

Our study showed similar operative and postoperative outcomes between supine XG-PCNL group with both prone and supine FG-PCNL groups. However, supine XG-PCNL was associated with higher puncture attempts, longer surgery duration, more upper and mid-pole punctures, and more nephrostomy tubes placed after PCNL procedure. The stone-free rate of the supine XG-PCNL was 85% and comparable to both supine and prone FG-PCNL groups. Some other studies also reported that PCNL under ultrasound guidance had high stone-free rate with low complication rate, and it was reported to be an effective and safe alternative to fluoroscopy when performed by experienced hands [4]. XG-PCNL has several advantages such as visualisation of nearby organs, avoidance of radiation exposure, and no requirement of using lead aprons compared to fluoroscopy guidance [3,13].

In this study, the baseline patient demographic characteristics among the groups were comparable. More patients had hydronephrosis (50%) in the supine XG-PCNL group. However, the differences were not significant when compared with those in both supine and prone FG-PCNL. Ng et al. [14] also found no difference in the hydronephrosis rate between the XG-PCNL and FG-PCNL groups. Despite its difficulty, Gamal et al. [15] reported that XG-PCNL can be safely performed in a non-distended collecting system. Li et al. [16] also presented 132 cases of successful puncture under ultrasound guidance after dilatation of an artificial retrograde collecting system. In our study, the collecting system was also dilated by maintaining continuous pump of normal saline via a ureteral catheter retrogradely by using pressure infusion bag. This demonstrated that XG-PCNL is a safe and reproducible technique, even in non-hydronephrosis kidney stone.

In our study, the number of puncture attempts was significantly higher in the supine XG-PCNL group, with a mean of 2.0 ± 1.7 times, than in the supine and prone FG-PCNL groups. In addition, the duration of surgery was significantly longer in the supine XG-PCNL group than in the FG-PCNL group. This result was different with that of Ng et al. [14] who found no difference in the operation duration between XG-PCNL and FG-PCNL. The higher puncture attempts and longer surgery duration in the present study might be attributed to the learning curve, since the XG-PCNL was a new procedure in our centre.

For puncture site, a significant difference was found between supine XG-PCNL and both FG-PCNL groups. There were more upper (25%) and mid-pole (42.5%) punctures in the supine XG-PCNL group, while majority of patients in the supine and prone FG-PCNL groups had lower pole puncture (87.5% and 80%, respectively). Some studies showed that upper pole puncture was correlated with increased risk of thoracic injury [14,17]. Despite the higher number of upper pole punctures in the supine XG-PCNL group, thoracic injury was not reported. Ng et al. [14] also reported more upper pole punctures in their XG-PCNL group with no pleural or lung injury. This might be attributed to the better visualisation of the renal calyx and surrounding structures by ultrasonography [14].

In this study, significant difference was found in the tube placement following the PCNL procedure. A significantly higher number of patients in the supine XG-PCNL group (42.5%) had standard PCNL using both nephrostomy tube and DJ stent. This might be attributed to our learning curve. Since these cases were our first 40 cases, safety measures were needed to avoid significant postoperative complications. Nephrostomy tube placement can maintain urinary drainage, prevent urinary extravasation, and tamponade the bleeding [18]. However, most of the nephrostomy tubes were removed on the second postoperative day. Our result was different with those in several studies. Ng et al. [14] observed that the size of the nephrostomy tube was smaller in their XG-PCNL group, and this might be due to the utilisation of colour Doppler ultrasound to avoid areas with dense vascularisation [14].

In terms of outcomes, the difference in stone-free rate, complication rate, and postoperative length of stay among the groups were not statistically significant. The stone-free rate of supine XG-PCNL in our study was 85%. These results were consistent with those in several studies that found no significant difference and comparable stone-free rate in the XG-PCNL group [13,14,19,20]. Our result was also consistent with the finding of a meta-analysis by Yang et al. [4] Our postoperative length of stay was also comparable with those in several studies where the patients were usually discharged in the third postoperative day [3,20].

In our study, no significant complication was recorded during the procedure. Only postoperative fever was recorded. Other studies did not found any significant difference in the complication rate between XG-PCNL and FG-PCNL [3,14,19,21]. However, a meta-analysis by Yang et al. [4] found that XG-PCNL had significantly lower complication rate (OR 0.56, *p* = 0.009) than FG-PCNL. This may be due to the increased visibility of surrounding organs during percutaneous access in the XG-PCNL group.

Although there were more blood transfusions (25%) after supine XG-PCNL, this number was not significant. However, several other studies had lower blood transfusion rate in the XG-PCNL group [10,15]. The higher blood transfusion rate in our series might be caused by the lower preoperative haemoglobin level in our patients at approximately 12 g/dL. Therefore, lower blood loss might cause haemoglobin level to drop below 10 g/dL. In our centre, haemoglobin level of < 10 g/dL and symptomatic anaemia were indications of blood transfusion after a PCNL procedure. In several other studies, the patients had higher preoperative haemoglobin level than the level in our patients [3,13,22]. Akman et al. [22] also showed that preoperative haemoglobin level was a significant predictive factor affecting blood transfusion requirement in both univariate and multivariate analyses.

This study has several limitations. Although the patients in the XG-PCNL group were collected prospectively, the comparison groups, which were composed of patients who underwent supine and prone FG-PCNL, were historical control groups identified from our PCNL database. However, the inclusion and exclusion criteria were the same, and the baseline characteristics among the groups were comparable. Since supine XG-PCNL by using Alken metal telescoping dilators is a relatively new approach, further randomised clinical trial is needed to compare its effectiveness and safety to FG-PCNL. However, the result of our study can be used as basic data before conducting a randomised clinical trial. In our study, the PCNL procedures were also performed by two different main surgeons. However, both surgeons had similar technique, good experiences, and comparable skills.

## Conclusion

Supine XG-PCNL is an alternative to both supine and prone FG-PCNL with similar efficacy and complication rates for kidney stone patients. This could be a good alternative in urological centres with no access to fluoroscopy. However, our current initial practice was still associated with higher puncture attempts, nephrostomy tube placement, and longer surgery duration. As a novel procedure, with improved experience and learning curve, satisfactory results will be expected.

## Data Availability

The datasets analysed during the current study are available from the corresponding author on reasonable request.
